# Acquired infection after intubating patients with COVID-19: Datasets

**DOI:** 10.1016/j.dib.2020.106130

**Published:** 2020-08-06

**Authors:** Mingyang Sun, Ningtao Li, Xiaoyan Suo, Zhongyuan Xia, MingZhang Zuo, Hui Zhi, Renyu Liu, Jiaqiang Zhang

**Affiliations:** a Department of Anesthesiology and Perioperative Medicine, Henan Provincial People's Hospital, People's Hospital of Zhengzhou University, Zhengzhou, Henan 450003 China; b Department of Anesthesiology, Renmin Hospital, Wuhan University, Wuhan 430060, China; c Department of Anesthesiology, Beijing Hospital, National Center of Gerontology, Institute of Geriatric Medicine, Chinese Academy of Medical Sciences, Beijing 100730 China; d Department of Anesthesiology and Critical Care, Perelman School of Medicine at the University of Pennsylvania, 336 John Morgan Building, 3620 Hamilton Walk, Philadelphia, PA 19104, USA

**Keywords:** COVID-19, Airborne precaution, Acute respiratory distress syndrome, Intubation, Airway management

## Abstract

Thirty-six anesthesia departments in 36 hospitals in four provinces of China where an outbreak of COVID-19 occurred were surveyed. We found that there were ten anesthesiologists (5 male and 5 female) who contracted the infection after performing intubation, as well as 4 nurses (1 male and 3 female) who contracted the infection after assisting with the intubation. This is a retrospective investigation and no intervention was applied.

The numbers are presented as mean ± Standard Deviation (SD). We used Graphpad Prism (version 8.2.1 Windows version, GraphPad Software, San Diego). Fisher's exact test at a two-sided significance level of 0.05 was used to identify potential risk factor (s) for intubation providers. A *P* value less than 0.05 is considered statistically significant. A total of 211 anesthesiologists from four provinces were involved in the intubation of 664 patients with confirmed or potential COVID-19. Of these 644 patients, 640 cases were eventually confirmed with a diagnosis of COVID-19. Among the 211 anesthesiologists who performed intubation, 10 of them had a confirmed diagnosis of COVID-19 afterwards. Coughing is a risk factor for provider infection (*P* = 0.0001). The number of intubation attempts (within three attempts) did not increase the risk of the infection. All of the affected anesthesiologists had symptoms 2–12 days after the intubation encounter (average 6 ± 3 days). All had radiological image evidence of bilateral pneumonia and all reported relatively mild symptoms. The affected doctors were out of clinical service for 20–60 days (average 46 ± 12 days). Seven of the doctors have been discharged from the hospital, but three of them remain hospitalized. Four nurses who assisted with intubations contracted COVID-19. One of these nurses was in critical condition but was eventually discharged with a loss of 50 days of clinical service. The remaining three nurses have had mild symptoms so far, but one is still hospitalized.

**Specifications Table**

**Table:** 

**Subject**	Anesthesiology and Pain Medicine
**Specific subject area**	Physician safety
**Type of data**	Table
**How data were acquired**	Survey Electronic questionnaire
**Data format**	Raw:Excel Analyzed: Table
**Parameters for data collection**	The data include factors that may contribute to COVID-19 infection during intubation by the intubator, for example: Department, Doctor's gender, Doctor's title, Doctor's age, years of working as an anesthesiologist, Intubate directly for the patients with COVID-19, Patient diagnosed with COVID-19 before intubation, Patient diagnosed with COVID-19 after intubation, Prevention measures, Induction method of anesthesia, Anesthetics, Bucking or not, Intubation device, How many times was the intubation successful? How many days suffered from COVID-19 infection after intubation? Current status, Quarantines measures, Recovery or not, The days out of clinical practice, Whether family members and colleagues are infected.
**Description of data collection**	The electronic questionnaires (Appendix I) were sent to the directors of Anesthesiology departments of 36 hospitals in 4 provinces. The 36 directors of Anesthesiology departments asked the secretaries to fill in the questionnaires according to the contents of the questionnaires and the conditions of the department staff. The first questionnaire was sent out at the end of February 2020 to departmental chairs in 36 hospitals in four provinces where an outbreak of COVID-19 occurred, to identify information about intubation for COVID-19 patients in January and February 2020. Medical providers who contracted COVID-19 were also identified. After we identified the physicians who may have contracted the disease after performing intubation, a second questionnaire (Appendix II) was sent out to these individuals for more detailed information related to their infection and well-being for a detailed case analysis including reviewing the medical record for the intubation event. Only those with a confirmed diagnosis of COVID-19 (positive computed tomography scan for an atypical bilateral pneumonia and positive viral test) were included.
**Data source location**	Institution: The hospitals of Hubei Province City/Town/Region: Hubei Province Country: China Institution: The hospitals of Henan Province City/Town/Region: Hunan Province Country: China Institution: The hospitals of Zhejiang Province City/Town/Region: Zhejiang Province Country: China Institution: The hospitals of Hunan Province City/Town/Region: Hunan Province Country: China
**Data accessibility**	The data are published along with the article.
**Related research article**	Jiaqiang Zhang, Mingyang Sun, Ningtao Li, Xiaoyan Suo, Zhongyuan Xia, Mingzhang Zuo, Renyu Liu. Acquired infection after intubating patients with COVID-19: A retrospective study, J Clin Anesth 2020, In press.

**Value of the Data**

• The novel coronavirus disease (COVID-19) is very contagious and was declared a pandemic by the World Health Organization on March 11, 2020. These data provide critical evidence regarding how many doctors were infected by COVID-19 at the time of intubation, and the risk factors for infection [1].
• These data tell us that the highest level of protection for airborne infectious disease (Class III) should be adopted by all persons involved in intubation, and that muscle relaxants should be required for intubating COVID-19 patients. The doctors who provide intubations for patients with COVID-19 can benefit from these data [1].
• These data tell us that medical providers who perform intubations for COVID-19 patients have a high risk of contracting the disease, ranging from 1.56% to 4.37%. The highest level of protection (Class III) and muscle relaxant should be used during intubation of COVID-19 patients.

## 1 Data description

The data include 1 table and 1 supplemental file containing a questionnaire relevant to the table. The questionnaire was designed by the research team (Table 1).

**Table 1:** Characteristics of the infected medical providers.

Medical provider number	Gender	Age	working experience (year)	Diagnosis before intubation	Diagnosis after intubation	Protection Level	Quarantine/ isolation	Discharged	Absent clinical days	Family member infection
1	Male	30s	>10	Yes	Yes	III	Yes	Yes	50	No
2	Male	30s	>10	No	Yes	II	No	Yes	41	Yes
3	Male	30s	>10	Yes	Yes	III	No	Yes	50	Yes
4	Female	30s	9	No	Yes	I	Yes	Yes	30	No
5	Female	30s	7	No	Yes	III	Yes	Yes	50	No
6	Male	40s	20	Yes	Yes	I	Yes	Not yet	20	No
7	Female	40s	25	No	Yes	I	Yes	Not yet	50	Yes
8	Male	30s	11	No	Yes	I	Yes	Yes	60	No
9	Female	40s	>10	Yes	Yes	I	Yes	Yes	50	No
10	Female	30s	>10	Yes	Yes	I	No	Not yet	60	Yes
11	Male	30s	18	No	Yes	I	Yes	Yes	50	No
12	Female	20s	5	No	Yes	I	Yes	Not yet	50	Yes
13	Female	30s	11	Yes	Yes	I	Yes	Yes	30	No
14	Female	30s	8	Yes	Yes	I	Yes	Yes	30	No

All of the affected anesthesiologists had symptoms 2–12 days after the intubation encounter (average 6 ± 3 days). All of them were out of clinical service for 20–60 days (average 46 ± 12 days). Seven of the 10 affected anesthesiologists have been discharged from the hospital; three of them are still hospitalized. Three of the four affected nurses have been discharged, and one is still hospitalized after 50 days. The average days out of clinical service were 40 ± 11 days. Of the 14 affected providers, only 3 had used Class III precautions, 10 of them used Class I precautions, and 1 used Class II precautions.

## 2 Experimental design, materials and methods

Two electronic questionnaires were designed to investigate the risk of infection when anesthesiologists perform endotracheal intubation on COVID-19 patients. The first questionnaire was sent out at the end of February 2020 to departmental chairs in 36 hospitals in four provinces, where an outbreak of COVID-19 occurred, to identify information about intubation for COVID-19 patients from in January and February 2020. Medical providers who contracted COVID-19 were also identified. The first survey questionnaire to identify providers who were potentially infected after performing intubation. After we identified the physicians who may have contracted the disease after performing intubation, a second questionnaire (the questionnaire is provided as a supplementary file) was sent out to these individuals to obtain more detailed information related to their infection and well-being, as well as a detailed case analysis including review of the medical record for the intubation event. Only those with a confirmed diagnosis of COVID-19 (positive computed tomography scan for an atypical bilateral pneumonia and positive viral test) were included. We also asked these physicians whether there were other persons who assisted with these intubations. Additional information about the affected persons who assisted with intubations was also obtained and analyzed. The level of protection and precaution used for infectious disease is provided as Appendix III (the questionnaire is provided as a supplementary file). The numbers are presented as mean ± Standard Deviation (SD). We used Graphpad Prism (version 8.2.1 Windows version, GraphPad Software, San Diego). Fisher's exact test at a two-sided significance level of 0.05 was used to identify potential risk factor (s) for intubation providers.

## Ethics statement

This is a retrospective survey. We assure that the work described has been carried out in accordance with The Code of Ethics of the World Medical Association. The informed consents were obtained. The privacy rights of human subjects were always observed. The survey will not cause any harm or adverse effects for subjects.

## Declaration of Competing Interest

All of the authors declare that they have no known competing financial interests or personal relationships which have, or could be perceived to have, influenced the work reported in this article.

## Appendix I

### Questionnaire 1

Please answer the following questions with your best effort.

How many male patients with suspected or confirmed COVID-19 were intubated by physicians in your department in January and February 2020?
How many female patients with suspected or confirmed COVID-19 were intubated by physicians in your department in January and February 2020?
Among all the above patients, how many tested positive for COVID-19 with a confirmed diagnosis?
How many physicians in your department participated in intubation for patients with COVID-19?
How many of them used Class I precaution?
How many of them used Class II precaution?
How many of them used Class III precaution?
Did your hospital or department have an adequate amount of Powered Air Purifying Respirators?
How many patients were intubated with muscle relaxant?
How many patients were intubated with spontaneous breathing?
How many patients did not receive any muscle relaxant for intubation?
How many intubations were performed using video laryngoscope?
How many intubations were performed using regular laryngoscope?
How many intubations were performed using light wand?
How many intubations were performed using fiberoptic intubation?
How many difficult airways did you encounter?
How many had cough during intubation?
How many successful intubations were achieved with the first try?
How many successful intubations were achieved with the second try?
How many successful intubations were achieved with the third try?
How many failed intubations did you encounter?
How many doctors are infected after being involved in intubation for COVID-19?
How many doctors have tested positive with a confirmed diagnosis of COVID-19 after the intubation for COVID-19?
How many doctors had image evidence indicating pneumonia after the intubation for COVID-19?

## Appendix II

### Questionnaire 2

**Please answer the following questions with your best effort.**

What is your age, gender, and years of practice in anesthesiology with intubation experiences?
Was the patient diagnosed with COVID-19 before your intubation event?
Was the patient diagnosed with COVID-19 after your intubation event?
What kind of protective measures were taken?
What kind of induction mode was used?
What medications were used for induction?
Did the patient have cough during intubation?
What kind of intubation tool (laryngoscope) was used?
How many attempts for a successful intubation?
How many days after the intubation event did you start to have symptoms?
What is your severity of symptoms?
Were you under quarantine or isolation after the intubation event?
Are you discharged from hospital?
How many clinical service days did you lose?
Have any of your family members had COVID-19 after your intubation event?
Have any of your assistants had infection after the intubation event?
If yes, could you please provide the following information?

• The nurse's gender, age and number of years of practice in anesthesia
• Quarantine/isolation after intubation
• The severity of the symptom
• Whether recovered or still hospitalized
• Days out of clinical service

## Appendix III

Definition of the Class of precaution used in China

Class I precaution

• Strictly follow the standard prevention principle
• Strictly follow sterilization and isolation policy
• Wear work cloth, work hat and surgical face mask, isolation gown, and latex gloves when necessary
• Strict hand hygiene
• Personal hygiene after event: pay special attention to airway and mucosal protection

Class II precaution

• Strictly follow the standard prevention principle
• Strictly follow sterilization and isolation policy
• Wear work cloth, work hat and surgical face mask, wear isolation gown, and wear shoe cover when necessary
• Wear gloves when contact body fluid, secretions, urine and feces
• Wear goggle and face shield when have potential projectile objects or event
• Strictly follow the area regulations, proper donning and doffing the personal protective equipment. Pay special attention to airway, oral and nasal mucosal protection, and eye protection

Class III precaution

• Strictly follow the standard prevention principle
• Strictly follow sterilization and isolation policy
• Wear work cloth, work hat and surgical face mask, wear isolation gown, and wear shoe cover when necessary,
• Medical-grade Powered Air Purifying Respirator (PAPR) should be used when necessary
• Wear gloves when in contact with body fluid, secretions, urine, and feces
• Wear goggle and face shield when have potential projectile objects or event
• Strictly follow the area regulations, proper donning and doffing the personal protective equipment; pay special attention to airway, oral and nasal mucosal protection, and eye protection
